# Electrooculograms for Human–Computer Interaction: A Review

**DOI:** 10.3390/s19122690

**Published:** 2019-06-14

**Authors:** Won-Du Chang

**Affiliations:** School of Electronic and Biomedical Engineering, Tongmyong University, Busan 48520, Korea; chang@tu.ac.kr; Tel.: +82-51-629-1314

**Keywords:** electrooculogram (EOG), bio-signal processing, EOG calibration, eye-tracking

## Abstract

Eye movements generate electric signals, which a user can employ to control his/her environment and communicate with others. This paper presents a review of previous studies on such electric signals, that is, electrooculograms (EOGs), from the perspective of human–computer interaction (HCI). EOGs represent one of the easiest means to estimate eye movements by using a low-cost device, and have been often considered and utilized for HCI applications, such as to facilitate typing on a virtual keyboard, moving a mouse, or controlling a wheelchair. The objective of this study is to summarize the experimental procedures of previous studies and provide a guide for researchers interested in this field. In this work the basic characteristics of EOGs, associated measurements, and signal processing and pattern recognition algorithms are briefly reviewed, and various applications reported in the existing literature are listed. It is expected that EOGs will be a useful source of communication in virtual reality environments, and can act as a valuable communication tools for people with amyotrophic lateral sclerosis.

## 1. Introduction

In the past decades, keyboards, mice, and touchscreens have been the most popular input devices to control computers. These traditional devices are effective in most general situations such as for writing documents, searching and navigating the internet, and gaming. However, people may encounter special situations in which such traditional devices are not effective, for instance, when people want to operate computer devices in a hands-free manner. Such scenarios may include surgery, as medical doctors often wish to search for information and discuss the procedure with advisors during an operation [1]; augmented reality (AR) applications, as they require fast and intuitive interactions [2]; or some gameplaying [3,4]. Some people also experience difficulties in using traditional input devices. Furthermore, people with advanced amyotrophic lateral sclerosis (ALS) are unable to communicate with people except by using their eye movements [5,6].

Numerous studies have been performed focusing on novel input approaches for such situations. Hand-movements were detected using optical or infrared cameras, and the intention of a user was recognized [7,8]. Bioelectric signals, such as electroencephalograms (EEGs), electromyograms (EMGs), and electrooculograms (EOGs) were also utilized to understand a user’s intention [9,10,11].

Among these approaches, the present paper focuses on the approach of utilizing EOGs. An EOG originates from the standing potential between the front and back of the eyes, and thus, it represents eye-movements [12,13]. The use of EOGs was one of the first methods to measure eye-movements [14], and it has been expected to be a promising source for communications between human and computers [11,15,16]. 

The main benefit of utilizing EOG as an input source of human–computer interaction (HCI) is that the eye-movements can be estimated using low-cost devices. It is known that a complete EOG recording system can be assembled for less than 100 EUR [17], and eye-movements can be estimated with a precision of up to 1.5° [18]. Considering this background, several researchers have investigated EOG-based HCI systems. Although most of these studies were focused on utilizing instant movements in a single direction, recent studies on eye-writing have enabled the estimation and recognition of more complex eye-movements [16,19,20], thereby allowing users to write Arabic numbers, English alphabets, and Japanese Katakana directly. In this manner, the users can communicate even complicated messages in a relatively short time.

Several review papers on eye tracking exist, in which the origin and measurements methods of EOGs are briefly introduced. Young and Sheena reviewed early techniques for measuring eye movements in 1975 [14,18]. They compared the techniques of electrooculography, corneal reflexes, and contact lenses. Techniques on tracking the limbus, pupil, and eyelids have also been reviewed. In the perspective of EOG, early historical aspects and measurement methods have been described well. A comprehensive review on eye tracking was conducted in 1995 by Glenstrup and Engell-Nielsen in their bachelors’ thesis [21], in which eye tracking techniques, psychological and physiological aspects concerning eye tracking, and relevant applications in literature were described in detail. However, because their review was focused on a wide research domain, studies on EOGs were not detailed adequately. Singh and Singh reviewed eye tracking technology including EOGs in 2012 [22]. They described a concise history of eye tracking and types of eye movements, and compared four approaches of eye tracking: scleral search coil, infrared oculography, electro-oculography, and video oculography. The basic characteristics of EOGs—such as the origin of the signal, types of electrodes, and a typical electrode placement—were also introduced briefly.

The purpose of this study is to summarize the recent studies on EOGs and suggest research directions in the aspect of HCI. In spite of recent advances on this topic—EOGs for HCI—there have not been many attempts to summarize the recent progresses. Previous reviews have briefly introduced EOGs, but have not covered recent advances. By summarizing the recent advances, I would like to create a small guide for studying this topic, as many different methods on measuring and signal processing exist. The aims of this study are as follows: (1) to suggest a generalized procedure for utilizing EOGs, (2) to categorize and discuss methods in previous studies at each step of the generalized procedure, and (3) to suggest a research direction for the topic. This paper describes the characteristics of EOGs, measurement issues, and signal processing techniques. The presented contents would be valuable in developing EOG-based applications or initiating research on this topic.

## 2. Characteristics

**Figure 1:**
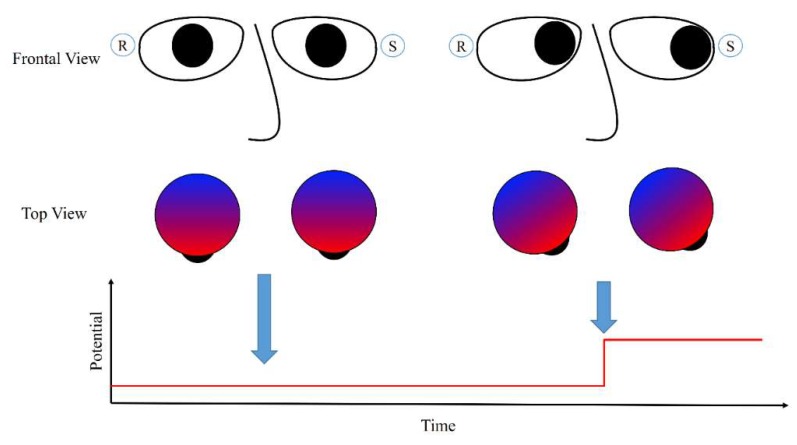
Illustrations of eye-movements and changes in electric potential around the eyes. S denotes the location at which a sensor is placed to record the EOG signal, and R is the location of a reference electrode.

An EOG is a signal that changes according to eye-movements; the changes are caused by the standing potential between the retina and cornea of the eyes [12,23]. The potential increases when the cornea approaches an electrode, and it decreases when the cornea moves in the opposite direction. Figure 1 illustrates this process. When the eyes are directed straight and the gaze is fixed, the electric potential does not change and remains at a certain value. When the eyes move left to the sensor, the electric potential increases sharply as the cornea reaches the sensor. This phenomenon also occurs when the eyes are closed [24], or in the case of blind people [25].

An EOG often reflects activities other than eye-movements, and these are commonly named artifacts. Eyelid-related artifacts are strong and occur frequently when closing and opening the eyes. Such artifacts correspond to a dramatic increase in the potential above the eyes. This high potential is the sum of the retinal and corneal potentials, as the eyelid conducts the positive charges of the cornea to the frontopolar region when it comes in contact with the cornea [23]. Hence, although eyelid-related artifacts originate from the same source as that of the eye-movement signals, it is not significantly related to the eye-movement: The eyes move only slightly (less than 5°) during eye blinks as there exists a mechanism to suppress saccadic movements during eye blinks [23,26]. 

EMGs represent another artifact in EOGs, and they are observed when a person moves his/her facial muscles or body during EOG measurement. EMGs originate from activities such as jaw clenching, raising of the eyebrow, smiling, or walking [27,28,29]. The shapes of these artifacts differ according to the types of movement, electrode position, and sampling rates [11,27,28]; however, generally, high-frequency noise and plateau-like waveforms, which exceed the potential range of eye-movement-related signals, are noted [11,29]. The shapes of a facial EMG measured above the eyes are illustrated well in Ref. [27]. 

## 3. Overall Procedure of Utilizing EOGs for Human–Computer Interface

**Figure 2:**
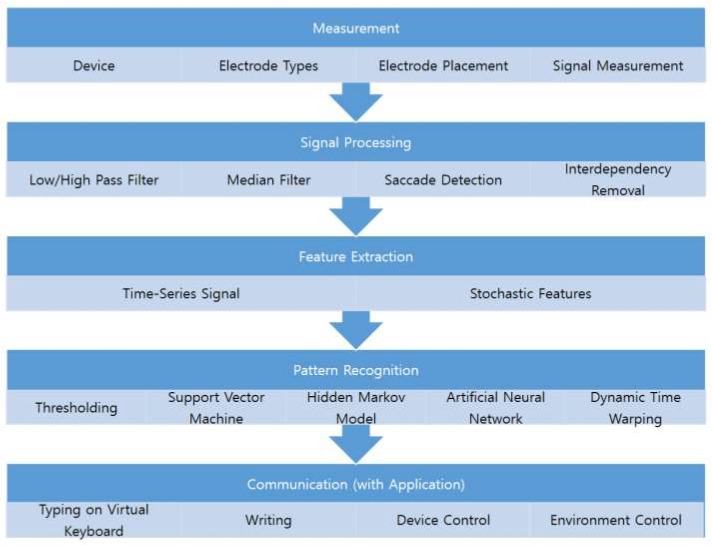
Overall Procedure of Utilizing EOGs for Human–Computer Interface.

The experimental procedures reported in the literature differ according to the purpose of the study, however, they can be generalized into five steps: measurement, signal processing, feature extraction, pattern recognition, and communication (see Figure 2). These steps are not mandatory, and may be omitted depending on the purpose, signal quality, and applications. For example, the raw signal could be directly used for training a pattern recognition model.

The first step is measurement, during which researchers need to decide the type of device to be used for data acquisition and select the types and placement of electrodes. The decision made in this step determines the initial quality of the signal and affects the later steps. The device can be stationary or mobile; the stationary devices are typically more expensive and support more accurate bioelectrical signal measurement. The electrode placement may affect the signal quality [30]. In Section 4, I summarize and discuss the electrode placement used in previous studies.

Signal processing is the next step, in which the eye-related signals are extracted and separated. Because EOGs are composed of signals from various origins, it is important to remove signals not related to the eyes. EMGs, drifts, and high frequency noises are well-known signals that need to be removed from the EOGs; the removal methods vary in the literature. Crosstalk between EOG channels are also signals that need to be removed, which have not been discussed much in the past. These signals are suppressed and partly removed at this step, and further removed in the pattern recognition step. The methods and a discussion of this issue are summarized in Section 5.

The processed signal must be classified after feature selection. The feature selection is designed in consideration of recognition tasks and models to train. A typical feature for majority of the recognition tasks is the processed signal of the previous step, in which the series of signals are considered as an input vector for a recognition model. The hidden Markov model (HMM) and dynamic time warping (DTW) based approaches often consider these features [16,20]. Stochastic features, such as the mean of saccadic duration, fixation rates, and blink rates, have been used for the support vector machine (SVM) and artificial neural network [20,31,32]. 

Recognition algorithms are widely available, and the SVM, HMM, artificial neural network, and DTW techniques have been utilized to recognize EOG signals, although the most popular algorithm is the thresholding method. The recognition algorithms have been utilized for both recognizing users’ intentions and filtering out EMG artifacts or eye blinks, where the users’ intentions are expressed by single or complex eye movements. The thresholding methods are popular in classifying the single directional movements, and advanced algorithms, such as the SVM, HMM, and DTW have been utilized for classifying the complex eye movements [16,20,25,31]. Although recognition algorithms for EMG artifacts have not been studied much yet, detection of eye blink signals has been widely studied because eye blink removal is an important issue in EEG signal processing in the prefrontal region of the brain [33]. Thresholding amplitude is a popular algorithm to detect the eye-blink region in signals, and template matching algorithms with DTW, correlation coefficients, and other dissimilarity features have been proposed in the past decades [34]. Finally, the classified activity of a user is transferred into target systems; this activity could be used to control other devices or environments, type using a virtual keyboard, or directly write alphabets. The subsequent sections review previous studies and discuss each of these steps.

## 4. Measurement

**Figure 3:**
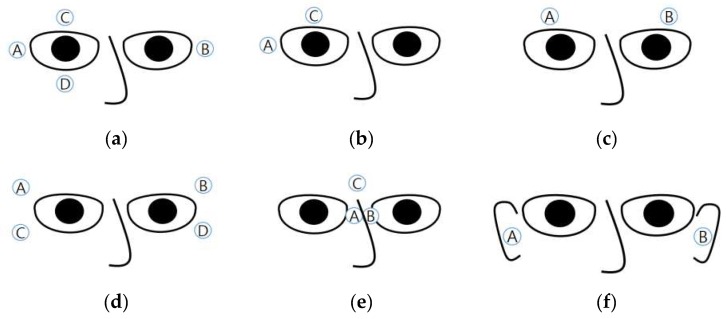
Six different electrode positions (from **a** to **f**) to measure EOGs in the literature. The letters A, B, C, and D indicated in circles denote the locations of electrodes.

The measurement of an EOG typically involves placing sensors at the left and right sides of the eyes (for horizontal EOG), and above and below an eye (for vertical EOG) [22,35]. Figure 3a illustrates the positions of the electrodes. The horizontal EOG is obtained by subtracting the signal pertaining to B from that of A (or that of A from that of B), and the vertical EOG is obtained by subtracting the signal pertaining to D from that of C (or that of C from that of D). The reference electrode is often placed on the forehead [15,16,31,36,37], in the middle of the eyes [38], or on the mastoids [35,39]. Young and Sheena suggested using six electrodes placed above and below both the eyes of the user [14].

Some studies used only two electrodes in addition to a reference electrode: One above one eye and the other to the right of the right eye (or to the left of the left eye) (Figure 3b) [25,40,41]. Expectedly, the signals obtained using this electrode placement were less stable than those obtained using other methods; however, it was possible to recognize eye movements in eight directions with an accuracy of over 80% [25].

Some atypical electrode placements are shown in Figure 3c–f. The placement in Figure 3c involves embedding electrodes in a headband [27,42], which may increase the usability of the measuring device. Under this placement, the vertical eye movements are ignored. Yan et al. proposed a method of placing electrodes above and below the right side of the right eye and electrodes above and below the left side of the left eye (Figure 3d) [30]. Twenty-four levels of eye movements (−75° to 75° horizontal movements) were successfully classified with an accuracy of 87.1%, but a comparison between electrode placement was not performed. Kanoh et al. proposed the measurement of EOGs by using three electrodes—two in the middle of the eyes and one between the eyebrows [43]. The electrodes were embedded at the nose-pads (A and B in Figure 3e) and on the bridge of the eyeglasses (C in Figure 3e). The horizontal EOG was obtained by subtracting the signal pertaining to A from that of B, and vertical EOG was obtained by subtracting the signal pertaining to (A+B)/2 from that of C. A product with this electrode placement was eventually commercialized by a Japanese company. This product enabled the measurement of EOGs just by wearing glasses, thereby improving its usability. Figure 3f shows another electrode placement. Favre-Felix et al. proposed the use of in-ear electrodes to record horizontal EOGs [44]. The accuracy of in-ear EOGs was slightly worse than that of conventional EOGs (correlation of 82.60% compared to correlation of 90.11% pertaining to conventional EOGs), although this accuracy could be improved by implementing advanced signal processing algorithms.

The electrode placements illustrated in Figure 3c,e,f were designed to be embedded in mobile devices. The quality of the obtained signal still remains unproved. Studies on the electrode placement and quality of the EOG signal would be necessary for future research.

The EOG amplitude of eye-movement is typically less than 500 μV [35], and the amplitude is proportional to the magnitude of eye movements [37]. Yagi et al. confirmed this by performing 70 min recordings per participant [45]. However, it is difficult to utilize a small amount of eye movements owing to DC drifts and other noises [18]. More DC drift is incurred as the recording time increases, and because of this, most applications utilize a large amount of eye movements or consider movements performed in a short time period [11,16,20].

**Figure 4:**
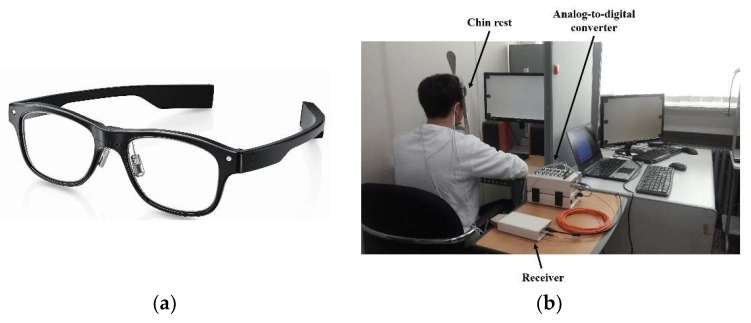
Devices with different styles and types of electrodes. (**a**) Dry electrodes embedded in a mobile device [52], (**b**) a typical stationary device with wet electrodes [35]. Reprinted with permission.

An EOG is recorded using an amplifier because its amplitude is of the order of microvolts. Several types of measurement systems exist. The devices typically used for electroencephalograms (EEGs) can be utilized for EOG recording. Stationary EEG recording devices commonly cost over 10,000 EUR (BioSemi [11,20,35], NeurOne [39,46], BioPac [47,48], and NF Corporation [41,49]). Figure 4b shows a recording environment with a stationary system (BioSemi). Some companies offer recording systems designed for EOGs (which record two to four channels of bioelectric signals). These include BlueGain [16,50], NI-USB-6008 [27,51], and JinsMeme [43,52], which cost from 300 to 1600 EUR. Among these, JinsMeme is a mobile system, in which an EOG amplifier is embedded in eyeglasses (Figure 4a). BioPac also offers a mobile device to record EOGs, in which the device is connected to the stationary system (MP160) via wireless communication [48]. Many researchers also introduced self-designed EOG systems [15,29,36,53,54,55,56,57,58]. Some of these systems are implemented as stationary types, which are connected to a computer via a cable [36,57], whereas the others have portable forms, in which the devices are installed in a wheelchair [15,59] or embedded into goggles [29,56,60]. 

In the literature, the use of wet electrodes is popular. This is possibly because many studies did not focus on making a product, as signal to noise ratios (SNRs) of the wet electrodes are higher. However, glasses with dry electrodes have demonstrated impressive performance comparable to that of wet electrodes. Bulling et al. classified six eye-movement activities (copying, reading, writing, watching a video, browsing, and no activity) with the use of dry electrodes leading to an accuracy (F1 score) of 68.5 [31], and they also reported the classification of eight eye-gestures with an accuracy of 87% [29]. Lledó et al. recognized six-directional eye-movements with an accuracy of 90% [60]. Although direct comparisons between the types of electrodes have not been carried out yet, the recognition accuracies do not exhibit a considerable difference. However, it can be expected that the noises caused by body movements or sweat degrade the quality of the signal more when dry electrodes are used compared to that in the case of wet electrodes, because the contact between the electrodes and skin could worsen in mobile environments. 

## 5. Signal Processing

The main purpose of EOG signal processing is to remove noise and emphasize the signal of interest. A number of different eye movement types exist: saccades (fast eye movement), fixation (fixing gaze onto a subject), miniature eye movements (drift, tremor, and microsaccades during fixation), smooth pursuit (when tracking a slowly moving target), and vergence (inward and outward eye movements) [22]. Among these movement types, saccades and smooth pursuit have been the main research source of signal for HCI in the past decades, as the others, except for fixation, are uncontrollable. Hence, these eye-movement related signals have been considered as noise to remove in this field.

High frequency noises, such as tremor or high-frequency EMG artifacts, have been often removed using median and low-pass filters [11,15,16,20,27,29,38,53,54,55,57]. The cut-off frequency for the low-pass filters, as reported in the literature, varies from 10 to 60 [15,16,27,38,53,54,55,57]. There is no consensus on the best filter to remove these noises, but the use of low-pass filters has been preferred in the literature. It is visually observable that a low-pass filter does not preserve the edge steepness of saccadic eye movements [56], and using a median filter, ten eye gestures could be recognized with an accuracy of 95.93% [20]. In addition, Bulling et al. demonstrated that an adaptive median filter is superior to a low-pass filter [56]. However, a recent study employed the median filter together with a 20-Hz low-pass filter, and twelve eye gestures were reported to be recognized with an accuracy of 95%; further, it was claimed that a cut-off frequency of 20 Hz is sufficient to include intentional eye-movements and remove high-frequency noises [16]. Despite this slight contradiction, both methods could obtain applicable results. Hence, as noted in [39], it is reasonable to consider that the method for high frequency removal should be selected taking into account other procedures of the entire system.

**Figure 5:**
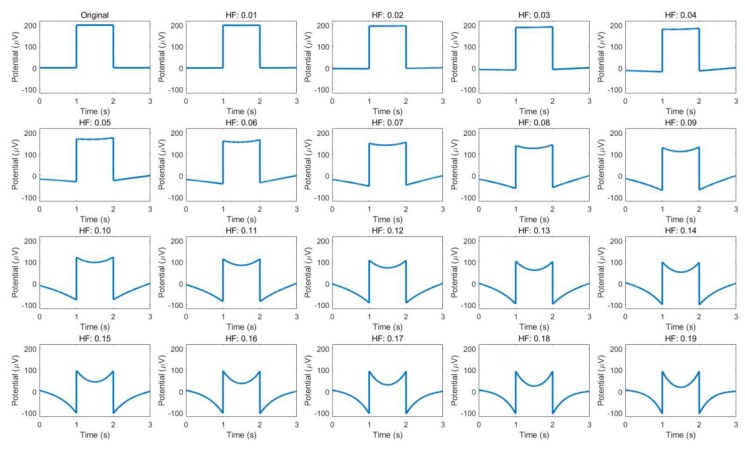
Artificially generated eye movement signal and the changes after applying high-pass filters of different cut-off frequencies. The cut-off frequencies are noted above each axis, while the original signal is displayed in the top-left.

Drift has conventionally been removed using high-pass filters, with the cut-off frequency varying from 0.05 to 0.1 [15,38,53,54]. In a few exceptional cases, cut-offs of 0.175 [61] and 0.53 Hz [40] were used; however, these cases dealt only with single and directional eye movement [40], and they utilized the high-pass filtered signal together with the raw signal [61]. The reason for the low variation is that the low-pass filter easily deforms the signal during saccade and fixation. Figure 5 illustrates how the signals are deformed by high-pass filters as the cut-off frequency increases. An ideal eye movement signal is noted to be generated (top-left) and the high-pass filtered signal with a different cut-off frequency is displayed. The experimental code was written and run using Matlab^TM^ and the first-order Butterworth filter was utilized, as in [34,57]. It is clearly shown that during fixation, the signal becomes rounded, which can be easily confused with the signals pertaining to smooth pursuit or complex and continuous movements.

To avoid this problem, some researchers attempted to utilize alternative methods for drift removal. Polynomial regression is one of these methods [11,39], in which the EOG signal is fit to a polynomial expression and the fitted signal is subtracted from the original signal. The degree of the polynomial varies from 1 to 20, depending on the shape of the original signal. After removing the drift, Pettersson et al.’s method could detect horizontal and vertical saccades of eyes moving more than 7.5° with a sensitivity of 94% [39]. Another substitute for high-pass filtering is wavelet transformation. Bulling et al. proposed a method named CWT-SD, in which wavelet coefficients were calculated and signals were reconstructed for cases in which the coefficients were greater than a given threshold [29]. Pettersson et al. also removed high frequency noise over 100 Hz using the wavelet transformation [39]. The merits of this method were that the signals in the fixation region remained unchanged, and gradual changes such as drift were removed. This method was replicated by another research group, and they were able to recognize eye gestures after noise removal [11,20]. A disadvantage of utilizing the wavelet transformation is that slow eye movements such as smooth pursuit or the movements in a short distance are removed with drift. This could be an issue when an application is developed for people with ALS, because their eye movements could be slower than those of people without ALS [62]. 

Methods concerning the detection of eye blinking are described in Section 7. Typically, the eye blink artifact region is completely removed and the signal before and after the blink is interpolated [20,29], because although eye blinking occurs rarely during saccades, it occurs immediately before and after the saccades [29].

**Figure 6:**
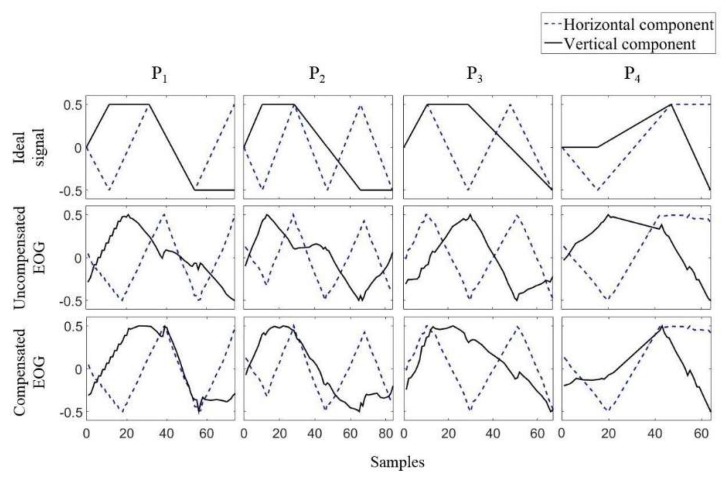
Crosstalk in EOG signals [35]. The horizontal component represents the EOG recorded at the left and right side of the eyes, whereas the vertical component represents the EOG recorded above and below an eye. The ideal signal is the expected EOG when a participant moves his/her eyes as instructed. The compensated EOG is the signal after the crosstalk is removed from the obtained (uncompensated) signal.

The last feature that needs to be considered in the signal processing of EOGs is crosstalk, or the interdependency between the horizontal and vertical channels. In 2008, Tsai et al. [63] reported that a large amount of horizontal movement led to signal changes in vertical EOGs. The first strategy for addressing crosstalk was to ignore EOG signals with low amplitude. In 2016, another research group raised this issue and studied it intensively [35]. The authors instructed participants to move their eyes according to dots on a screen, and the EOGs during the experiments were recorded. By analyzing the experimental data, the authors reported that crosstalk occurred in the case of nine out of ten participants, and the amount of crosstalk was different for each participant. The authors also proposed a simple method to remove this crosstalk, which involved first measuring the amount of crosstalk by recording the EOG during horizontal eye movement, and then removing the amount of crosstalk for the test signals. Examples of the ideal EOG signal, measured signal, and compensated signal are shown in Figure 6.

## 6. Applications for Human–Computer Interface

HCI applications for EOGs are often developed for paralyzed people, such as people with ALS. People suffering from ALS gradually lose the ability to control their muscles, and they lose most of their controls in the later stages of this disease [5,6]. Because eye-movements are one of the ultimate ways of communication in such scenarios, it could be valuable if an EOG-based communication tool is developed as a product for patients and their caregivers.

Another application field of EOGs is gaming. In a virtual reality (VR) game environment, it is not easy to use conventional input devices. Voice recognition technology is a powerful tool and has demonstrated high accuracies; however, it could disrupt the player’s surroundings.

Most HCI applications of EOG reported in the literature utilize two signal sources: saccades and eye blinks. Saccades are often used for controlling movement, and blinks help in selections. This section lists recent applications utilizing EOGs for HCI. Please note that although I classified eye blink as an artifact according to the EOG’s definition in Section 2, the eye blink is an important signal that can be obtained from EOGs because the eye blink is distinguished from eye-movements in EOGs. The eye blink can be utilized for selecting a button or starting an eye-based communication system. 

**Figure 7:**
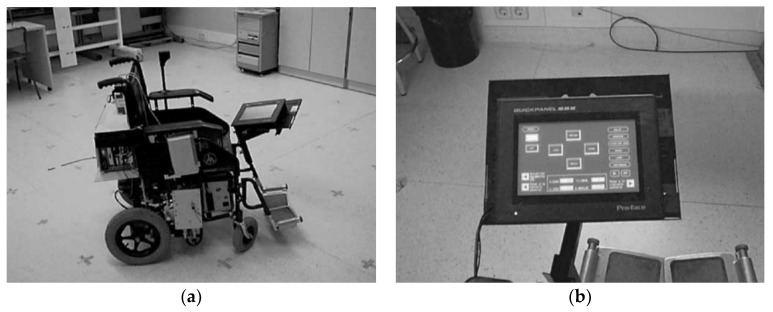
Wheelchair embedded with EOG input interface. (**a**) Overlook (**b**) Control display. Reprinted with permission from Ref. [15].

In 1999 and 2002, Barea et al. proposed a wheelchair with an EOG input interface [15,59]. A user could control the wheelchair by providing six different commands to the wheelchair by using his/her eye movements: forward, backward, left, right, stop, and speed control. Four directional movements were commanded using fast and long saccadic movements of upward, downward, leftward, and rightwards. The speed was controlled via small and gradual eye movements (see Figure 7 for the wheelchair and menu screen). The stop condition was activated when the eyes were closed. In a comparable manner, some researchers suggested the use of EOG communication for robot control. Rusydi et al. and Oh et al. independently suggested moving a robot according to the moving direction and angle of the user’s eyes [41], and to control a robot’s moving direction and speed by using eye movements in four directions [42]. The studies of both Rusydi et al. and Oh et al. suggested the idea of the application without implementation.

Some studies suggested utilizing eye-movements for cursor control. Yan et al. introduced a mouse control system for answering a questionnaire [64]. The system allows a user to select the answer by moving a cursor in a 4 × 4 grid, after displaying a question for a few seconds. The user can move the cursor by moving the eyes in four directions. The cursor does not move when the electric potential caused by an eye movement is smaller than the threshold. Deng et al. implemented a remote controller for a TV, in which the four-directional movements corresponded to changes in the channel and volume [53]. The channel changed when the eyes moved vertically, and the sound volume increased/decreased when the eyes moved horizontally. 

**Figure 8:**
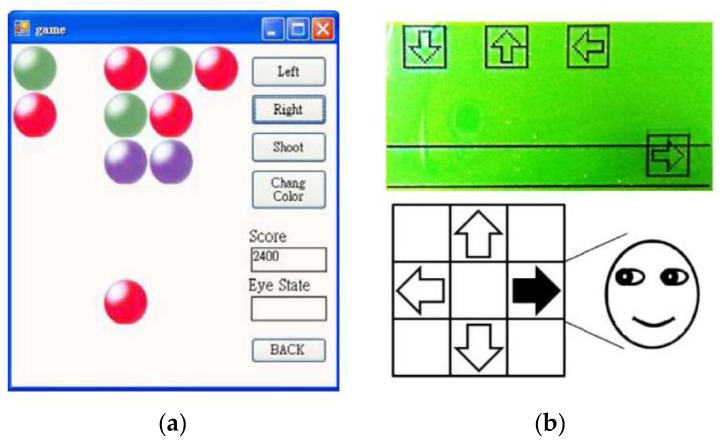
Games controlled using eye movement. (**a**) Shooting game to find the same color [53]. (**b**) Dance Dance Revolution game in which the user moved his/her eyes to follow directional instructions [38]. Reprinted with permission.

Games are another application of utilizing EOG. A simple shooting game was proposed in the same study conducted by Deng et al. [53]. The goal of the game was to shoot the same colored balls as that of the user’s ball, in which the user’s ball moved left and right via eye movements (Figure 8a). The user’s ball at the bottom, as illustrated in Figure 8a, moves left and right according to the eye movements, and the user can change the ball color by moving the eyes downwards. Target balls were displayed in a 3 × 5 grid, in three different colors. The user can shoot the ball by moving the eyes upwards, and the target balls having the same color as that of the user’s in the same line disappear. The game finished when all the target balls disappeared.

Kim and Yoon developed a Dance Dance Revolution game with eye movements [38]. The user was required to move his/her eyes by following directional instructions on the game screen, in which arrows for the instruction dropped gradually from top to bottom (see Figure 8b). The user was required to follow the instructions before the arrows reached the bottom; the game was terminated otherwise. It was difficult to recognize directions when the users repeatedly moved his/her eyes toward the same direction. They amplified the signal sufficiently and set a threshold for noise removals precisely. 

Kumar and Sharma [65] implemented a VR game with EOG, named “VRailSurfer.” In this game, the user controls a game character’s movement through eye blinks. The goal of the game was to survive and collect coins during the run on the rails. The game character was required to avoid trains by moving left or right, and jumping; the movements of the character were controlled by blink, double blink, and wink, respectively. The accuracy of the blink detection and VR control were 96% and 80%, respectively. The drop in the accuracy for VR control was caused by impedance changes during the head movement, although wet and sticker-type electrodes were used.

Although majority of the previous studies have focused on utilizing a limited number of commands, such as the four-directional movements, there have been a few attempts to enable keyboard typing or writing with EOGs. Yamagishi et al. proposed a method for a user to type letters [25]. A virtual keyboard in which cursors could move in eight directions according to eye movements was introduced. The users could freely move the cursor over a keyboard on a screen with eye movements, and wink to select the letter on the cursor. The merit of this system was that it facilitated complex communication. Instead of choosing one of four predefined options, users could communicate freely, similar to when using a computer keyboard.

Xiao et al. proposed a typing system with eye blink [66]. The system displayed letters in 4 × 8 grids, in which each button flashed in a random order. A user was required to stare at a button to type and blink when it flashed. The system recognized the button by synchronizing the flashes and detecting the eye blink. The user can type a letter in 2 s using this system, as the flashing duration is as short as 100 ms and there is an overlap of 40 ms between two adjacent flashes. This was an impressive idea, which enabled fast communication using eye blinking only. 

**Figure 9:**
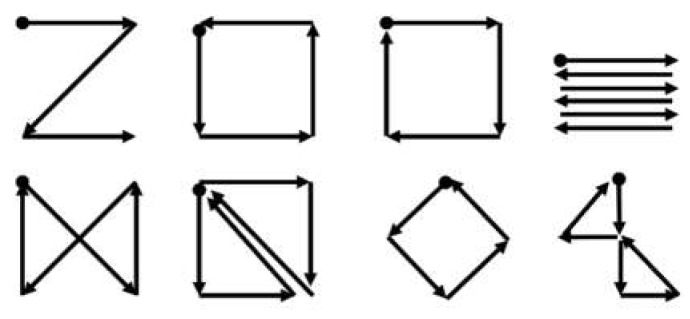
Eye gestures of different complexities [29]. Dots denote the starting points.

The gesture recognition proposed by Bulling et al. could be utilized in a similar way [29]. In their study, eight different gestures—combinations of eye movements in different directions—were designed and recognized (see Figure 9). These eye gestures could provide users with more options to communicate, especially because these gestures were diverse and easy to remember.

In this perspective, eye-writing—in which users draw letters directly using eye movements—is a meaningful approach for utilizing EOGs for HCI. The concept of eye-writing was first introduced by Tsai et al. in 2007 [63].

Ten Arabic numbers and four mathematical symbols were written by 11 users, and fairly acceptable accuracy was achieved (believability: 75.5%, dependability: 72.1%). The authors also applied the same system to 40 different letters (English alphabets, Arabic numbers, and punctuation symbols). The reported mean accuracy was 80% for five users, although the small number of participants was a limitation of the study.

**Figure 10:**
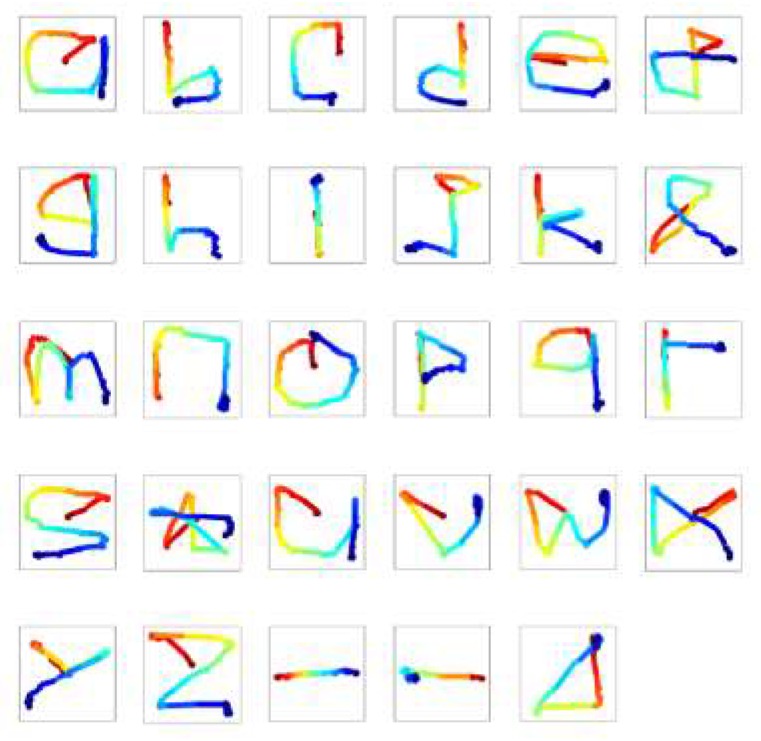
Example of eye-written characters (26 English alphabets and three punctuation symbols). The color of traces indicates the stroke order (from red to blue). Reprinted with permission [11].

In 2016, Lee et al. attempted to build a system of eye-writing English letters [11]. They made participants sit in front of an empty panel, and eye-write letter by letter according to audio instructions. The stroke orders of 12 letters (c, e, f, i, j, o, r, t, y, space, backspace, and enter) were predefined to avoid confusion, as the shapes of some letters are remarkably similar in the absence of pen-up information. The participants were asked to write naturally, in the manner in which they hand wrote letters. Figure 10 shows a set of the eye-written letters. The accuracy (F1 score) of the system was 87.38% for 20 participants. 

Recently, in 2018, Fang et al. published an article on eye-writing for Japanese Katakana [16]. Because Katakana is mostly composed of straight lines, the researchers developed a system to recognize 12 basic types of strokes. By recognizing these strokes, the proposed system was able to classify all the Katakana (48 letters). The accuracy of Katakana recognition was 93.8% for six participants. One of the distinguishing features of the study was that continuous eye-writing was implemented. By ignoring small eye movements, the system could recognize the eye-writing of multiple letters continuously without discrete sessions. The mean input rate was 27.9 letters per minute.

**Figure 11:**
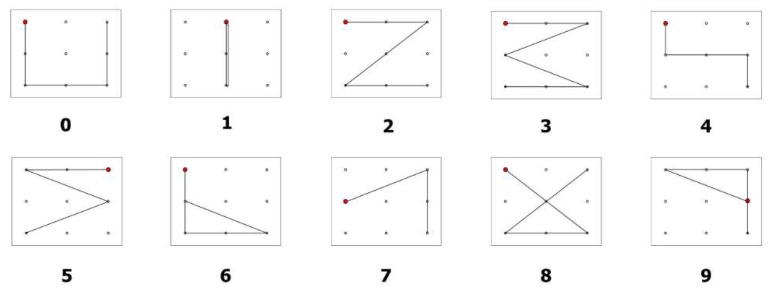
Design of eye-writing digits for individuals with ALS. Reprinted with permission [20].

The study of Chang et al. also considered eye-writing, but it focused on the feasibility of applying the eye-writing technique to individuals suffering from ALS [20]. They proposed a novel design of ten digits (from 0 to 9) so that people may eye-write with minimum effort. The shapes of the digits were designed to be similar to the corresponding Arabic numbers, to be short as possible, and distinguishable from other digits (see Figure 11). Eye-writing experiments were conducted with 18 healthy participants (healthy group) and three participants with ALS (ALS group). Because people with ALS get fatigued easily and they find it difficult to communicate verbally, all the instructions for eye-writing were explained systematically. The mean accuracies of the healthy and ALS groups were 95.74 and 86.11, respectively. Despite the reduction in the mean accuracy, the study verified that the EOG-based HCI system could be used as a communication tool for people with ALS.

## 7. Feature Extraction and Pattern Recognition

Pattern recognition algorithms play the role of interpreting EOG signals for a given practical use. The algorithms are required for the following tasks: eye blink detection, EMG detection, recognition of saccadic direction, and eye-writing/activity recognition. Feature extraction is necessary to minimize the complexity of recognition problems. By converting the raw signals into notable features, higher accuracy may be achieved.

Automatic detection of eye blink artifacts has been studied extensively in the past decades. One of the easiest methods for such detection is to determine a threshold and classify a region of the signal as an eye blink if the potential of the region is higher than the threshold [67]. This method has an advantage when high accuracy is not necessarily required [68], as it can be easily implemented in a short time. The main limitations of this method are the relatively low accuracy for detection and low precision for determining the range of the artifacts because the detection was conducted epoch by epoch, that is, it was determined if an epoch included an eye blink [34]. The mean accuracy of the amplitude thresholding was 93.24% for 24 participants when an advanced method achieved 96.02% for the same database [34]. The drop in the accuracy was mainly caused by the variation of the eye blink amplitudes among participants.

To detect eye blink with better accuracy, advanced algorithms have been utilized. Chambayil et al. utilized the kurtosis of adjacent windows, and minimum and maximum amplitude of signals as input features to determine if a signal in a window includes an eye blink [32]. A multi-layer perceptron model was developed with nine hidden layers. The reported regression value was 84.99% for 384 epochs from 13 participants. Delorme et al. suggested a method of utilizing kurtosis and entropy of a signal for eye blink detection [69]. The authors found that the epochs with eye blinks exhibit higher kurtosis and entropy, and these features were utilized to classify the epochs with eye blinks after applying independent component analysis (ICA). Durka et al. utilized the correlation coefficients between the EEG channels [70]. An epoch was said to include eye blinks, if the coefficient between the EEG signals at Fp1–F3 and F3–C3 or at Fp2–F4 and F4–C4 is higher than the threshold. The accuracy of the proposed system was similar to that of the human experts: the area under curve (AUC) of the proposed system was 91.5% whereas the AUC of human experts was 95.4%. Chang and Im suggested utilizing dynamic positional warping—a template matching method that identifies the corresponding points dynamically [71]. The authors achieved an accuracy of 87.13%, which was higher than that of the traditional dynamic time warping (77.92%). The authors also proposed a method to determine the eye blink region without employing templates by utilizing the summation of the derivatives within multiple windows (MSDW) [34]. The reported mean accuracy was 96.02% for the detection of epochs contaminated by eye blink artifacts, and the system detected more than 80% of the artifact region with an accuracy of 87.96%. 

The automatic adaption of a system to a user is another issue in eye blink detection [39,72]. Pettersson et al. proposed a method of selecting a threshold to distinguish saccadic or eye blink from noise [39]. The method first determines all the local maxima and sorts them according to the amplitudes. Subsequently, the curve formed by the maxima’s amplitudes is fit to two consecutive straight lines, where the joint point becomes the threshold. Chang et al. proposed a similar algorithm by utilizing the histogram of the local maxima [72]. A notable point of this study is that the authors detected eye blinks in real time while adjusting the threshold. The proposed algorithm determined a user’s personal threshold for detecting eye blinks in a few seconds.

The detection or removal of EMG artifacts from EOG has not been studied extensively yet. Most studies on EOGs involve controlling the users’ body or facial movements, and focus on clean eye-movement-related signals [16,20,35]. Bulling et al. suggested utilizing an adaptive median filter to remove EMGs from EOGs [29]. The adaptive filter reduced EMGs during a walk by up to 80% in horizontal EOGs and 60% in vertical EOGs. The window size of the median filter is critical and different for each activity. The window size was adjusted to cover each activity by utilizing an accelerometer.

A few studies classified facial EMGs from the obtained EOG signals. Lee et al. considered a signal region with a higher amplitude than that of the typical EOG signals, which was experimentally set to 800 μV [11]. Paul et al. classified EOGs and EMGs by using different bandpass filters (10 Hz low-pass filter for EOGs, and 25–125 Hz for EMGs) [27]. The signals with a larger rooted mean sum (RMS) than the threshold were considered as EMGs, in which the threshold was set to 120 mV through calibration.

Classifications of eye-written characters and eye activities have utilized elaborate algorithms. Tsai et al. utilized an artificial neural network (learning vector quantization) for eye-writing recognition [63]. The input features were the initial movement direction and the number of directional changes in horizontal and vertical EOGs. The proposed system recognized 14 patterns (10 Arabic numbers and four arithmetic symbols) with an accuracy (believability) of 75.5%.

Lee et al. utilized DTW, which is a type of template matching technique [11]. Raw signals of horizontal and vertical EOGs were used as features after signal processing. The DTW technique involves finding the corresponding pairs between the template and test signal and calculating the dissimilarity between the two signals. Dynamic positional warping (DPW) is a modification of the conventional DTW, and is used to enhance the correspondence quality for two-dimensional data. Chang et al. classified Arabic digits with an accuracy of 95.74%, utilizing DPW together with the support vector machine (SVM) [20].

SVM is a traditional and popular classification model, which searches the hyperplane to maximize the distance between groups. This model was originally designed for binary classification, but it can solve multiple classification problems by utilizing multiple SVM models [73]. Bulling et al. utilized SVM solely to recognize six different activities (copying, reading, writing, watching a video, browsing, and no activity) [31]. Fifteen features were introduced to achieve a high accuracy, including the mean and variation of the amplitude of saccades, proportion of specific saccades, fixation rates, blink rates, and wordbook counts. The obtained accuracy (F1 score) was 73.19% when the verifications were conducted in a subject-independent manner. Bulling et al. also attempted to use the edit distance from string matching to recognize eight eye gestures (Figure 9). The eye gestures were similar to eye-writing, however, the traces of gaze did not form a letter. The attained accuracy was 87% in subject-dependent verification [29]. 

HMM is a method of probabilistic modeling for changes in states. Because this method is suitable for describing time-domain signals, it has been utilized for voice recognition, gesture recognition, and online handwriting recognition [74,75,76]. Fang et al. employed HMM together with a multilayer perceptron (MLP) to recognize ten basic strokes of Katakana [16]. The MLP was utilized to determine states in HMM, and HMM trained the state transitions and calculated the possibility of a series of transitions for a class. The MLP had two hidden layers with 200 and 100 nodes, respectively, and the trained HMM had 63.6 states on average for the user-independent model. The input features were raw signals filtered via a low-pass filter, delta, and delta-delta signals. They reported an accuracy of 86.5% for ten types of isolated strokes, which was 8.9% higher than the accuracy for dynamic time warping. 

After reviewing the pattern recognition algorithms for EOGs in the literature, sufficient evidence for the superiority of any algorithm could not be found. Although a number of studies compared their proposed method to conventional ones, the performance of an algorithm may differ according to the purpose of study, collected data, and employed signal processing methods. For intensive comparisons and advancement of this domain, I believe that in future studies, data will need to be collected considering various scenarios, and must be made public.

## 8. Limitations and Future Work

As described and discussed above, EOGs are an effective and cost-efficient way to estimate eye-movements. The approach involves recording eye movements under any light condition, and there is no influence from the presence of obstacles, even when the subject’s eyes are closed. Despite its merits, the number of studies on this subject is limited, and many topics and issues have been left unaddressed.

One of the most critical issues to utilize EOGs for HCI is the handling of artifacts, especially EMGs. Studies on EMG removal from EOGs are limited; however, more investigation is necessary. To increase usability, EOG-based HCI systems can be embedded into mobile devices by using dry electrodes. Another point to consider is that although mobile devices are easy for users or caregivers to carry, the mobile environment incurs unexpected movements of the users or devices, which generate EMGs or other artifacts. Future works on EOG-based HCI are expected to address this issue.

The next issue pertains to the application fields. Although the accuracy of the discussed systems was noted to be sufficiently high for practical use, the applications were limited to prototype games, wheelchair control, and virtual keyboards. I believe that the field of application must be expanded in the present scenario. Because a user can make four to eight selections in a few seconds, complicated systems for control devices could be invented in the near future. Eye-writing, which involves directly inputting characters by moving the eyes, is a relatively novel approach for EOG-based communication, and it could be embedded in virtual reality (VR) devices.

**Figure 12:**
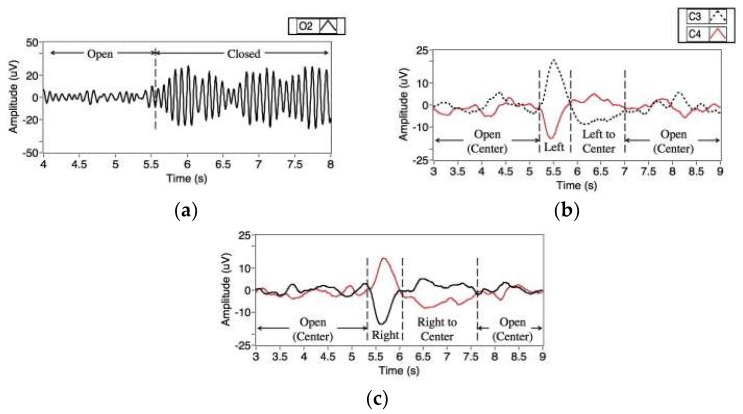
Eye- and eyelid-related signals observed at EEG channels. (**a**) Signal at O2 when closing and opening eyes, (**b**) Signals at C3 and C4 when moving eyes left, (**c**) Signals at C3 and C4 when moving eyes rights. Reprinted with permission [77].

A research topic for future study is a hybrid system with other biosignals for HCI. There are a number of hybrid systems in the literature, however, ways of utilizing these signals are several. Ramli et al. proposed a wheelchair control system with electroencephalograms (EEGs) [77]. They utilized signals from three EEG channels (C3, C4, and O2 in the ten-twenty electrode placement) and successfully classified EEG signals of four categories (eye closed and eye moving left, right, and center). This study demonstrated that placing electrodes around the eyes is not mandatory for the recording of EOGs. Moreover, it indicated the possibilities of utilizing other EEG features together with EOGs without placing additional electrodes on the user’s face. Figure 12 shows eye- and eyelid-related signals observed at EEG channels. Similarly, Oh et al. proposed a hybrid system utilizing both EEGs and EOGs, which classified eye closing/opening, and the movement of eyes in the left/right direction [42]. It was different from Ramli’s system in terms of the placement of electrodes: The EEGs and EOGs were measured from the prefrontal region in the proposed system. The authors confirmed that EEG measured from the prefrontal region could also support EOG-based HCI by recognizing if the eyes are open and closed automatically, thereby leading to an increase in the accuracy of the entire system.

Ma et al. proposed another hybrid system with EEG for robot control [78]. The system was composed of two different modes: EOG and EEG modes, where the modes switched by frowning. The EOG mode was used to control the directional movements of the robot and the EEG mode was used to control activities (receiving, passing over an object, sitting down, and dancing) or change the target robots. The EOGs were classified into seven activities (frowning, double and triple blink, left and right winks, and horizontal eye movements) using a thresholding based method. In the EEG modes, an event related potential (ERP) based system [79] was activated, in which a user was required to choose an icon from eight selections. 

By adding more sensors to record a user’s activities, an HCI system may increase the number of commands to control a device or increase the recognition accuracy of the activities. Minati et al. proposed a hybrid wearable device to control a robot arm, which measures EOGs, EEGs, EMGs, and accelerations. By utilizing multiple signals from a device, they increased the number and types of activities to command order to a system. The recognizable activities were head tilting, sideways head rotation, nodding, repeated or single eye blink, horizontal/vertical eye movements, jaw clenching, and brain activities in the alpha-band.

Another important research topic for future research is a standard database. Many research groups have introduced various methods, but it is difficult to compare and find the best method because of the lack of a common and standard EOG database. The standard database will help researchers to develop novel algorithms as the newly developed algorithms can be easily tested by the database.

## 9. Summary

This paper presents a review of the studies on EOGs from the perspective of HCI (the various studies are tabulated in Table 1). The topic—EOGs for HCI—has been widely studied in recent decades; however, a comparison between the studies has not been conducted enough. The main contribution of this study is that a general procedure for utilizing EOGs for HCI was suggested, and the recent studies were summarized for each step of the procedure. Through this study, researchers can overview the entire research field, and easily compare one study to another. Moreover, research directions can be derived from this study by reviewing the overall research progress in this field. A number of future research directions, which may inspire the readers are suggested.

EOGs are typically obtained around the eyes, by measuring the electric potential between opposite sides of the eyes. Commonly, four electrodes are used (one each above and below an eye, and to the left and right of the eyes), and the minimum number of electrodes is three (at both sides of the bridge of nose and between the eyebrows). This author found no evidence of any claim to substantiate the superiority of an electrode position against the others; however, the use of a smaller number of electrodes may increase the usability and reduce manufacturing costs.

A number of different signal processing and pattern recognition algorithms exist for EOGs. Artifacts and noises are required to be removed for further processing, and the movement directions, eye gestures, and eye-written characters are recognized by the algorithms. The process is generalized as follows: (1) high/low-pass filtering, (2) median filtering, (3) eye blink removal, (4) saccade detection, (5) feature extraction, and (6) pattern recognition. There may exist variations in this procedure according to the purpose, types of activities, and combination of algorithms used for an application.

In the literature, EOG-based communication has been applied for typing on a virtual keyboard, writing letters directly, moving a mouse, and controlling a wheelchair or a robot. Most applications utilized directional eye movements in a short time period, although a few exceptions utilizing complex eye gestures exist. When these gestures involve the forms of general letters (English alphabets, Arabic numbers, or Japanese Katakana), the activity is called eye-writing, which is a relatively new concept of utilizing eye-movement for communication. Eye-writing increases the users’ freedom by allowing them to write complex words or sentences directly. These gesture recognition techniques may improve the EOG-based communication by increasing the speed of communication.

There are several promising directions pertaining to EOG-based communication. First, EOG-based communication needs to be tested in mobile environments to expand its usability; in this case, artifacts and other noises are the significant issues in building a reliable system. Second, new application fields for utilizing EOGs must be developed. For instance, input devices for VR environments would be interesting for both users and researchers. Finally, the use of hybrid systems with other bio-signals could improve the performance of EOG-based communication.

## Figures and Tables

**Table 1:** Summary of recent studies on EOGs for human computer interface.

Categories	Types	References
Measuring Device	Stationary	[11,16,20,27,35,36,39,41,47,57,63]
	Mobile	[15,29,31,42,43,44,53,54,55,56,58,59,60]
Electrode Position *	a	[11,15,16,20,29,31,36,37,38,39,54,56,57,59,61,80]
	b	[25,40,41]
	c	[27,42]
	d	[30]
	e	[43]
	f	[44]
Signal Processing	Median filter	[11,16,20,29,31,35,56]
	Low/High pass filter	[15,16,25,27,37,38,40,53,54,55,57,59,61]
	Polynomial regression	[11,39],
	Saccade detection	[20,29]
	Interdependency removal	[35,63]
Feature	Time-series signal	[20,70,71,81]
	Stochastic feature	[32,69]
Pattern Recognition	Thresholding	[25,54,59,67]
	Support vector machine	[31]
	Hidden Markov model	[16]
	Artificial Neural network	[16,63]
	Dynamic time warping	[11,20,71]
Applications	Button selection	[61]
	Typing/ Virtual keyboard	[25,66]
	Eye-writing	[11,16,20,63]
	Robot control	[41,42,47]
	Wheelchair control	[15,59]
	TV control	[53]
	Game	[38,53,65]

* Refers to the number of electrode positions subfigure in Figure 3.

## References

[1] Muensterer O.J., Lacher M., Zoeller C., Bronstein M., Kübler J. (2014). Google Glass in pediatric surgery: An exploratory study. Int. J. Surg..

[2] Nilsson S., Gustafsson T., Carleberg P. (2007). Hands free interaction with virtual information in a real environment. PsychNol. J..

[3] Dorr M., Bohme M., Martinetz T., Brath E. (2007). Gaze beats mouse: A case study. PsychNol. J..

[4] Agustin J.S., Mateo J.C., Hansen J.P., Villanueva A. (2009). Evaluation of the potential of gaze input for game interaction. PsychNol. J..

[5] Beukelman D., Fager S., Nordness A. (2011). Communication support for people with ALS. Neurol. Res. Int..

[6] Communication Guide. http://www.alsa.org/als-care/augmentative-communication/communication-guide.html..

[7] Marin G., Dominio F., Zanuttigh P. Hand gesture recognition with leap motion and kinect devices. Proceedings of the 2014 IEEE International Conference on Image Processing.

[8] Liu H., Wang L. (2018). Gesture recognition for human-robot collaboration: A review. Int. J. Ind. Ergon..

[9] Lopes J., Simão M., Mendes N., Safeea M., Afonso J., Neto P. (2017). Hand/arm gesture segmentation by motion using IMU and EMG sensing. Procedia Manuf..

[10] Han C.H., Kim Y.W., Kim D.Y., Kim S.H., Nenadic Z., Im C.H. (2019). Electroencephalography-based endogenous brain-computer interface for online communication with a completely locked-in patient. J. Neuroeng. Rehabil..

[11] Lee K.-R., Chang W.-D., Kim S., Im C.-H. (2016). Real-time ‘eye-writing’ recognition using electrooculogram (EOG). IEEE Trans. Neural Syst. Rehabil. Eng..

[12] Malmivuo J., Plonsey R. (1995). Bioelectromagnetism: Principles and Applications of Bioelectric and Biomagnetic Fields.

[13] Frishman L.J. (2013). Electrogenesis of the electroretinogram. Retina.

[14] Young L.R., Sheena D. (1975). Survey of eye movement recording methods. Behav. Res. Methods Instrum..

[15] Barea R., Boquete L., Mazo M., López E. (2002). Wheelchair guidance strategies using EOG. J. Intell. Robot. Syst. Theory Appl..

[16] Fang F., Shinozaki T. (2018). Electrooculography-based continuous eye-writing recognition system for efficient assistive communication systems. PLoS ONE.

[17] Borghetti D., Bruni A., Fabbrini M., Murri L., Sartucci F. (2007). A low-cost interface for control of computer functions by means of eye movements. Comput. Biol. Med..

[18] Young L.R., Sheena D. (1975). Eye-movement measurement techniques. Am. Psychol..

[19] Tsai J.-Z., Chen T.-S. Eye-writing communication for patients with amyotrophic lateral sclerosis. Proceedings of the SIGACCESS Conference on Computers and Accessibility.

[20] Chang W.-D., Cha H.-S., Kim D.Y., Kim S.H., Im C.-H. (2017). Development of an electrooculogram-based eye-computer interface for communication of individuals with amyotrophic lateral sclerosis. J. Neuroeng. Rehabil..

[21] Glenstrup A.J., Engell-Nielse T. (1995). Eye Controlled Media: Present and Future State.

[22] Singh H., Singh J. (2012). Human eye tracking and related issues: A review. Int. J. Sci. Res. Publ..

[23] Iwasaki M., Kellinghaus C., Alexopoulos A.V., Burgess R.C., Kumar A.N., Han Y.H., Lüders H.O., Leigh R.J. (2005). Effects of eyelid closure, blinks, and eye movements on the electroenacephalogram. Clin. Neurophysiol..

[24] Schleicher R., Galley N., Briest S., Galley L. (2008). Blinks and saccades as indicators of fatigue in sleepiness warnings: Looking tired?. Ergonomics.

[25] Yamagishi K., Hori J., Miyakawa M. Development of EOG-based communication system controlled by eight-directional eye movements. Proceedings of the 2006 International Conference of the IEEE Engineering in Medicine and Biology Society.

[26] de Visser B.W.O., Bour L.J. (2006). Eye and Eyelid Movements during Blinking: An Eye Blink Centre?.

[27] Paul G.M., Cao F., Torah R., Yang K., Beeby S., Tudor J. (2013). A smart textile based facial EMG and EOG computer interface. IEEE Sens. J..

[28] Perdiz J., Pires G., Nunes U.J. Emotional state detection based on EMG and EOG biosignals: A short survey. Proceedings of the 2017 IEEE 5th Portuguese Meeting on Bioengineering (ENBENG).

[29] Bulling A., Roggen D., Tröster G. (2009). Wearable EOG goggles: Seamless sensing and context-awareness in everyday environments. J. Ambient Intell. Smart Environ..

[30] Yan M., Tamura H., Tanno K. (2014). A study on gaze estimation system using cross-channels electrooculogram signals. Int. Multiconf. Eng. Comput. Sci..

[31] Bulling A., Ward J.A., Gellersen H., Tröster G. (2011). Eye movement analysis for activity recognition using electrooculography. IEEE Trans. Pattern Anal. Mach. Intell..

[32] Chambayil B., Singla R., Jha R. EEG eye blink classification using neural network. Proceedings of the the World Congress on Engineering 2010.

[33] Jung T.-P., Makeig S., Westerfield M., Townsend J., Courchesne E., Sejnowski T.J. (2000). Removal of eye activity artifacts from visual event-related potentials in normal and clinical subjects. Clin. Neurophysiol..

[34] Chang W.-D., Cha H.-S., Kim K., Im C.-H. (2016). Detection of eye blink artifacts from single prefrontal channel electroencephalogram. Comput. Methods Programs Biomed..

[35] Chang W.-D., Cha H.-S., Im C.-H. (2016). Removing the interdependency between horizontal and vertical eye-movement components in electrooculograms. Sensors.

[36] Usakli A.B., Gurkan S. (2010). Design of a novel efficient humancomputer interface: An electrooculagram based virtual keyboard. IEEE Trans. Instrum. Meas..

[37] LaCourse J.R., Hludik F.C.J. (1990). An eye movement communication-control system for the disabled. IEEE Trans. Biomed. Eng..

[38] Kim M.R., Yoon G. (2013). Control signal from EOG analysis and its application. Int. J. Electr. Comput. Electron. Commun. Eng..

[39] Pettersson K., Jagadeesan S., Lukander K., Henelius A., Haeggström E., Müller K. (2013). Algorithm for automatic analysis of electro-oculographic data. Biomed. Eng. Online.

[40] Hori J., Sakano K., Saitoh Y. Development of communication supporting device controlled by eye movements and voluntary eye blink. Proceedings of the 26th International Conference of the IEEE Engineering in Medicine and Biology Society.

[41] Rusydi M., Sasaki M., Ito S. (2014). Affine transform to reform pixel coordinates of EOG signals for controlling robot manipulators using gaze motions. Sensors.

[42] Oh S., Kumar P.S., Kwon H., Varadan V.K. Wireless brain-machine interface using EEG and EOG: Brain wave classification. Proceedings of the Nanosensors, Biosensors, and Info-Tech Sensors and Systems.

[43] Kanoh S., Shioya S., Inoue K., Kawashima R. Development of an eyewear to measure eye and body movements. Proceedings of the Annual International Conference of the IEEE Engineering in Medicine and Biology Society.

[44] Favre-Felix A., Graversen C., Dau T., Lunner T. Real-time estimation of eye gaze by in-ear electrodes. Proceedings of the Annual International Conference of the IEEE Engineering in Medicine and Biology Society.

[45] Yagi T., Kuno Y., Koga K., Mukai T. Drifting and blinking compensation in electro-oculography (EOG) eye-gaze interface. Proceedings of the 2006 IEEE International Conference on Systems, Man and Cybernetics.

[46] Tesla Bittium NeurOne^TM^. https://www.bittium.com/medical/bittium-neurone.

[47] Wijesoma W.S., Wee K.S., Wee O.C., Balasuriya A.P., San K.T., Soon K.K. EOG based control of mobile assistive platforms for the severely disabled. Proceedings of the IEEE Conference on Robotics and Biomimetics.

[48] BioPac-Electrooculogram Amplifier. https://www.biopac.com/product/electrooculogram-amplifier/.

[49] NF Corporation. http://www.nfcorp.co.jp/english/index.html.

[50] BlueGain EOG Biosignal Amplifier. https://www.crsltd.com/tools-for-vision-science/eye-tracking/bluegain-eog-biosignal-amplifier/.

[51] National Instrument. http://www.ni.com/en-us.html.

[52] JINS MEME. https://jins-meme.com/en/.

[53] Deng L.Y., Hsu C.-L., Lin T.-C., Tuan J.-S., Chang S.-M. (2010). EOG-based human–computer interface system development. Expert Syst. Appl..

[54] Choudhury S.R., Venkataramanan S., Nemade H.B., Sahambi J.S. (2005). Design and development of a novel EOG biopotential amplifier. Int. J. Bioelectromagn..

[55] Ding Q., Tong K., Li G. Development of an EOG (electro-oculography) based human-computer Interface. Proceedings of the International Conference of the IEEE Engineering in Medicine and Biology Society.

[56] Bulling A. (2010). Eye Movement Analysis for Context Inference and Cognitive-Awareness: Wearable Sensing and Activity Recognition Using Electrooculography.

[57] Venkataramanan S., Prabhat P., Choudhury S.R., Nemade H.B., Sahambi J.S. Biomedical instrumentation based on electrooculogram (EOG) signal processing and application to a hospital alarm system. Proceedings of the 2005 International Conference on Intelligent Sensing and Information Processing.

[58] Banerjee A., Datta S., Pal M., Konar A., Tibarewala D.N., Janarthanan R. (2013). Classifying electrooculogram to detect directional eye movements. Procedia Technol..

[59] Barea R., Boquete L., López E., Mazo M. Guidance of a wheelchair using electrooculography. Proceedings of the 3rd IMACS International Multiconference Circuits, Systems, Communications and Computers.

[60] Iáñez E., Azorin J.M., Perez-Vidal C. (2013). Using eye movement to control a computer: A design for a lightweight electro-oculogram electrode array and computer interface. PLoS ONE.

[61] Yan M., Go S., Tamura H. (2014). Communication system using EOG for persons with disabilities and its judgment by EEG. Artif. Life Robot..

[62] Kaethner I., Kuebler A., Halder S., Kathner I., Kubler A., Halder S., Käthner I., Kübler A., Halder S. (2015). Comparison of eye tracking, electrooculography and an auditory brain-computer interface for binary communication: A case study with a participant in the locked-in state. J. Neuroeng. Rehabil..

[63] Tsai J.-Z., Lee C.-K., Wu C.-M., Wu J.-J., Kao K.-P. (2008). A feasibility study of an eye-writing system based on electro-oculography. J. Med. Biol. Eng..

[64] Yan M., Tamura H., Tanno K. Development of Mouse Cursor Control System using Electrooculogram Signals and its Applications in Revised Hasegawa Dementia Scale Task. Proceedings of the 2012 World Automation Congress.

[65] Kumar D., Sharma A. Electrooculogram-based virtual reality game control using blink detection and gaze calibration. Proceedings of the 2016 International Conference on Advances in Computing, Communications and Informatics.

[66] Xiao J., Qu J., Li Y. (2019). An electrooculogram-based interaction method and its music-on-demand application in a virtual reality environment. IEEE Access.

[67] Nolan H., Whelan R., Reilly R.B. (2010). FASTER: Fully automated statistical thresholding for EEG artifact rejection. J. Neurosci. Methods.

[68] Aarabi A., Kazemi K., Grebe R., Moghaddam H.A., Wallois F. (2009). Detection of EEG transients in neonates and older children using a system based on dynamic time-warping template matching and spatial dipole clustering. Neuroimage.

[69] Delorme A., Makeig S., Sejnowski T. Automatic artifact rejection for EEG data using high-order statistics and independent component analysis. Proceedings of the International Workshop on ICA.

[70] Durka P., Klekowicz H., Blinowska K., Szelenberger W., Niemcewicz S. (2003). A simple system for detection of EEG artifacts in polysomnographic recordings. IEEE Trans. Biomed. Eng..

[71] Chang W.-D., Im C.-H. (2014). Enhanced template matching using dynamic positional warping for identification of specific patterns in electroencephalogram. J. Appl. Math..

[72] Chang W.-D., Lim J.-H., Im C.-H. (2016). An unsupervised eye blink artifact detection method for real-time electroencephalogram processing. Physiol. Meas..

[73] Hsu C.-W., Lin C.-J. (2002). A comparison of methods for multiclass support vector machines. IEEE Trans. Neural Netw..

[74] Längkvist M., Karlsson L., Loutfi A. (2014). A review of unsupervised feature learning and deep learning for time-series modeling. Pattern Recognit. Lett..

[75] Gales M., Young S. (2008). The application of hidden Markov models in speech recognition. Found. Trends Signal Process..

[76] Wang X., Xia M., Cai H., Gao Y., Cattani C. (2012). Hidden-Markov-models-based dynamic hand gesture recognition. Math. Probl. Eng..

[77] Ramli R., Arof H., Ibrahim F., Mokhtar N., Idris M.Y.I. (2015). Using finite state machine and a hybrid of EEG signal and EOG artifacts for an asynchronous wheelchair navigation. Expert Syst. Appl..

[78] Ma J., Zhang Y., Cichocki A., Matsuno F. (2015). A novel EOG/EEG hybrid human-machine interface adopting eye movements and ERPs: Application to robot control. IEEE Trans. Biomed. Eng..

[79] Zhang Y., Zhao Q., Jin J., Wang X., Cichocki A. (2012). A novel BCI based on ERP components sensitive to configural processing of human faces. J. Neural Eng..

[80] Chang W.-D., Cha H.-S., Lee C., Kang H.-C., Im C.-H. (2016). Automatic Identification of Interictal Epileptiform Discharges in Secondary Generalized Epilepsy. Comput. Math. Methods Med..

[81] Chang W.-D.C.W.-D., Shin J.S.J. DPW Approach for Random Forgery Problem in Online Handwritten Signature Verification. Proceedings of the 2008 Fourth International Conference on Networked Computing and Advanced Information Management.

